# Preoperative computed tomography–guided localization of pulmonary ground‑glass nodules: a comparison of conventional and soft hook‑wires

**DOI:** 10.20452/wiitm.2024.17910

**Published:** 2024-11-07

**Authors:** Sheng‑Zhi Fan, Yu‑Yu Ma, Yan Sun, Hao Xu, Wei Chen

**Affiliations:** Department of Radiology, Xinyi People’s Hospital, Xinyi, China; Department of Radiology, Xuzhou Central Hospital, Xuzhou, China

**Keywords:** computed tomography, ground-glass nodule, hook-wire, localization, lung

## Abstract

**INTRODUCTION:**

Hook-wire (HW) localization is the most frequently employed approach for preoperative localization of pulmonary ground-glass nodules (GGNs); however, the relative outcomes of conventional and soft HW localization of GGNs remain poorly understood.

**AIM:**

This study sought to compare the safety and efficacy of preoperative computed tomography–guided conventional and soft HW localization of pulmonary GGNs.

**MATERIALS AND METHODS:**

Between January 2023 and June 2024, consecutive patients with pulmonary GGNs underwent conventional or soft HW localization prior to video-assisted thoracoscopic surgery. Safety and clinical efficacy of these 2 localization strategies were compared.

**RESULTS:**

In total, 88 patients underwent conventional HW localization of 95 GGNs, and 82 patients underwent soft HW localization of 88 GGNs. Technical success rates for the conventional and soft HW groups were 96.8% and 100%, respectively (P = 0.25), and the duration of localization was similar in both groups (mean [SD], 10.1 [4.5] vs 10 [5.9] min; P = 0.97). Complications were significantly more common in the conventional HW group than in the soft HW group (48.9% vs 32.9%, respectively; P = 0.04). Visual analog scale scores in the conventional HW group were significantly higher than those observed in the soft HW group (mean [SD], 4.6 [0.6] vs 3.4 [0.6]; P = 0.001). The rates of technical success for limited resection procedures were similar in both groups (96.8% vs 100% in the conventional and soft HW groups, respectively; P >0.99).

**CONCLUSIONS:**

Conventional and soft HW strategies can both effectively facilitate preoperative pulmonary GGN localization, but the soft HW approach has a more favorable safety profile.

## INTRODUCTION 

Lung cancer remains the leading global driver of cancer‑associated death.^1^^;^^2^^;^^3^ Adenocarcinomas comprise approximately 40% of all lung cancers, and early‑stage adenocarcinomas generally present as ground‑glass nodules (GGNs) on computed tomography (CT) scans.4 The advent of CT‑based lung cancer screening programs has contributed to rising rates of GGN detection. ^5^

Lung adenocarcinomas are primarily classified into invasive adenocarcinomas (IAs), mini‑invasive adenocarcinomas (MIAs), and adenocarcinomas in situ (AISs), with IA cases comprising roughly 52% of all GGNs.^4^^;^^5^^;^^6^ Limited wedge or segmental resection performed through a video‑assisted thoracoscopic surgery (VATS) approach can provide curative outcomes for MIA and AIS patients, with a 5‑year survival rate of 100%.^7^ Individuals with IA need to undergo lobectomy; however, limited VATS resection is generally performed first in such patients as a diagnostic procedure.^8^^;^^9^^;^^10^

Unlike solid pulmonary nodules, GGNs cannot be palpated during VATS.^10^ Preoperative CT‑guided localization strategies have thus been employed to overcome this issue. Hook‑wire (HW) localization materials are among the most widely used in this context, as they can be readily inserted into the lung parenchyma.^11^ A conventional HW is rigid; however, its use is associated with patient discomfort and high complication rates reaching 56%.^12^ Thus, soft HWs have been recently developed as an alternative to conventional HW materials.^13^ While there have been some reports describing the soft HW‑based localization of pulmonary nodules,^13^^;^^14^^;^^15^^;^^16^ studies focused specifically on GGNs are still lacking.

**Figure 1:**
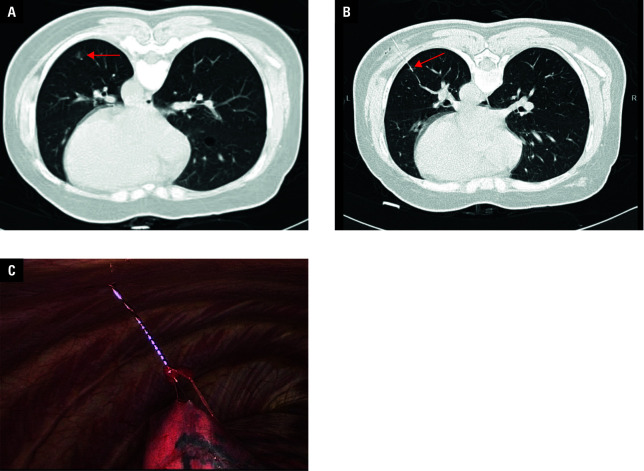
Periprocedural imaging of a patient undergoing computed tomography–guided conventional hook-wire (HW) localization; A – a ground -glass nodule (GGN) located at the right lower lobe (arrow); B – placement of the conventional HW near the GGN(arrow); C – conventional HW visualization during video-assisted thoracoscopic surgery

## AIM 

We sought to compare the safety and efficacy of preoperative CT‑guided conventional and soft HW localization of pulmonary GGNs.

## MATERIALS AND METHODS 

### Study design 

Between January 2023 and June 2024, consecutive patients with pulmonary GGNs scheduled for VATS resection underwent CT‑guided conventional or soft HW localization. The study inclusion criteria comprised 1) presence of pulmonary GGNs, 2) maximum long‑axis GGN diameter lower than or equal to 30 mm, and 3) age of 18 to 80 years. Patients were excluded if they 1) exhibited GGNs that were adjacent to vascular structures or the hilum, 2) exhibited GGNs below 6 mm in size, or 3) had any surgical contraindications. Indications for the surgical resection procedures for these patients included 1) recent increases in nodule size, 2) recent emergence or enlargement of solid nodule content, and 3) nodule solid content greater than or equal to 6 mm.

### Computed tomography–guided localization 

CT‑guided procedures were performed under local anesthesia. Patients were positioned according to the location of the target GGNs and the associated rib and vascular structures. Needle pathway selection was based on patient positioning, and the pathway with the shortest distance between the skin and the target GGN was chosen. 

In the patients undergoing conventional HW localization, a 20‑gauge, 10‑cm guiding needle (SOMATEX Medical Technologies, Berlin, Germany) was used for the procedure. The guiding needle was inserted into the lung parenchyma as per the selected needle pathway, and the positioning of the needle tip was confirmed via repeated CT scanning. When the needle tip was successfully located within 10 mm of the target GGN, the conventional HW was released Figure 1.

**Figure 2:**
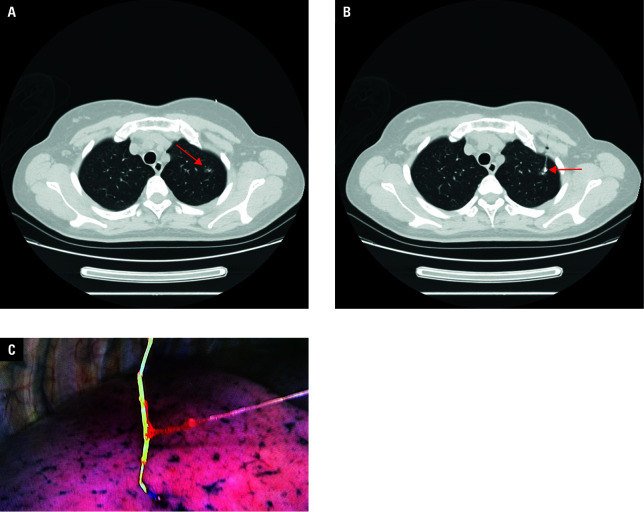
Periprocedural imaging of a patient undergoing computed tomography–guided soft hook-wire (HW) localization; A – a ground -glass nodule (GGN) located at the left upper lobe (arrow); B – placement of the soft HW near the GGN (arrow); C – soft HW visualization during video-assisted thoracoscopic surgery

In the patients undergoing soft HW localization, a 20‑gauge, 10‑cm guiding needle (Senscure, Ningbo, China) was used for the procedure. The needle puncture was performed in a manner identical to that adopted in the conventional HW group. During placement of the soft HW, its anchor was inserted proximal to the GGN. Subsequently, the needle was withdrawn in such a way that a tri‑colored suture connected with the anchor remained in the needle track and the distal end of the suture extended beyond the pleura Figure 2.

In the patients with multiple GGNs, a single‑stage procedure was used for localization of all target nodules. CT scanning was used to assess the patients for potential complications after localization.

### Video-assisted thoracoscopic surgery resection 

Limited VATS resection was performed within 3 hours of localization. All patients were placed in the lateral decubitus position, and a 3‑ to 5‑mm incision was made through the 4th or 5th intercostal space along the anterior axil‑lary line. The localization marker was initially visualized to confirm GGN localization, allowing surgeons to establish the position of the nodules based on a combination of GGN depth assessment on CT imaging and HW tip palpation. Wedge or segmental resection with a cutting suture device was then performed according to GGN depth. Segmental resection was chosen if the margin from the GGN edge exceeded 2 cm; otherwise, wedge resection was performed.

After resection, GGNs were subjected to rapid pathological examination. When the results indicated a diagnosis of IA, additional lobectomy and lymphadenectomy were performed. If MIA was diagnosed, lymph node sampling was conducted. No additional procedures were performed for GGNs classified as AIS or benign / precancerous lesions.

### Definitions 

Localization was considered successful if 1) the HW was visible during the VATS procedure, 2) no HW dislodgement occurred, and

3) the target GGN was present within the resected wedge / segment of the lung parenchyma. Duration of localization was defined as the interval between the first and the last CT scan. A visual analog scale (VAS; 0–10 points) was used to assess the severity of localization-related pain.

Technical success of localization was the primary study end point. Secondary end points included VAS scores, localization duration, localization‑associated complications, surgery type, and final patient diagnosis.

### Statistical analysis 

The normally distributed numerical variables were presented as mean (SD), and the non‑normally distributed variables were presented as median (interquartile range). Categorical data were presented as numbers and percentages. The independent‑sample t test or Mann–Whitney test was used to compare the numerical variables between the conventional and soft HW groups, while categorical data were compared with the χ2 test or Fisher exact test. A P value below 0.05 was considered significant. The SPSS 16.0 software (SPSS, Inc., Chicago, Illinois, United States) was used for all analyses.

### Ethics 

This retrospective study received approval from the Ethics Committee of Xinyi People’s Hospital (LL‑LW2024019), which waived the need for informed consent.

## RESULTS 

### Patients 

Between January and December 2023, a total of 88 patients underwent conventional HW localization of 95 GGNs (81 single GGN cases, 7 multiple GGN cases). Between January and June 2024, a total of 82 patients underwent soft HW localization of 88 GGNs (77 single GGN cases, 5 multiple GGN cases). Baseline patient data are presented in TABLE 1 .

Localization outcomes The outcomes of localization procedures are presented in TABLE 2 . The patients in the conventional and soft HW groups exhibited respective technical success rates of 96.8% and 100% (P = 0.25). Dislodgement was the cause of technical failure in all 3 unsuccessful cases in the conventional HW group. Duration of localization was similar in both groups (mean [SD], 10.1 [4.5] min vs 10.0 [5.9] min; P = 0.97).

**TABLE 1:** Baseline patient characteristics

Parameter		Conventional hook-wire group (n = 88)	Soft hook-wire group (n = 82)	*P *value
Age, y, mean (SD)		57 (10.1)	54.5 (12.8)	0.16
Sex	Men	33 (37.5)	21 (25.6)	0.1
	Women	55 (62.5)	61 (74.4)	
Number of nodules	Single	81 (92)	77 (93.9)	0.64
	Multiple	7 (8)	5 (6.1)	
Nodule diameter, mm, mean (SD)		10 (3.2)	8.5 (2.3)	0.001
Nodule-pleura distance, mm, median (IQR)		6.5 (2.7–14.5)	10 (3.3–19.5)	0.2
Nodule type	Mixed GGN	74 (77.9)	72 (81.8)	0.51
	Pure GGN	21 (22.1)	16 (18.2)	
Nodule location	Left lung	43 (45.3)	33 (37.5)	0.29
	Right lung	52 (54.7)	55 (62.5)	
Lung lobe	Upper	66 (69.5)	54 (61.4)	0.25
	Nonupper	29 (30.5)	34 (38.6)	

Data are presented as number (percentage) of patients unless indicated otherwise. Abbreviations: GGN, ground-glass nodule; IQR, interquartile range

**TABLE 2:** Localization-related data

Parameter	Conventional hook-wire group	Soft hook-wire group	*P *value
Successful localization, n (%)	92 (96.8)	88 (100)	0.25
Duration of localization, min, mean (SD)	10.1 (4.5)	10 (5.9)	0.97
VAS, points, mean (SD)	4.6 (0.6)	3.4 (0.6)	0.001
Complications, n (%)	43 (48.9)	27 (32.9)	0.04

Abbreviations: VAS, visual analog scale

Complication rates in the conventional HW group were higher than in the soft HW group (48.9% vs 32.9%, respectively; P = 0.04). Pneumothorax rates were 22.7% and 13.4% in conventional and soft HW groups, respectively (P = 0.12), and lung hemorrhage rates were 29.5% and 20.7% in conventional and soft HW groups, respectively (P = 0.19). Higher VAS scores were noted in the conventional HW group, as compared with the soft HW group (mean [SD], 4.6 [0.6] vs 3.4 [0.6], respectively; P = 0.001).

### Video-assisted thoracoscopic surgery outcomes 

The outcomes of the VATS procedures, including final diagnoses, are presented in TABLE 3 . Localization procedures that were a technical success were associated with successful limited VATS resection in all cases. Additionally, in 2 of the 3 patients who experienced a technical failure of localization, resection was successful as bleeding from the needle puncture site was still visible in the visceral pleura during VATS resection. In the remaining patient lobectomy was performed. Consequently, the respective technical success rates for limited resection were 96.8% (94/95) and 100% (88/88) in the conventional and soft HW groups (P >0.99). In the conventional HW group, 63 and 31 patients underwent wedge and segmental resection, respectively. In the soft HW group, wedge and segmental resection was performed in 67 and 21 patients, respectively.

## DISCUSSION 

CT‑guided localization has emerged as a routine approach to the preoperative management of patients undergoing VATS resection of pulmonary GGNs.^17^ Approximately 70% of patients with pure GGNs exhibit a pathological stage below IA.^6^ Accurate localization can help reduce the resected range and maximally preserve pulmonary function in these patients.

Several types of localization materials have been developed to localize GGNs,^18^ the most common of which being HW, coil, and liquid materials. Liquid‑based materials are inexpensive, safe, and easy to deploy; however, they are prone to diffusion, which may lead to localization failure. Also, they can only be used for localization on the pleural surface, and do not enable the assessment of GGN depth. The process of coil localization is complex owing to the coil shape and the requirement that its distal end be deployed next to the GGN, with its proximal end extending beyond the visceral pleura.^16^ The use of coils also entails a risk of the material being fully inserted into the lung parenchyma. In such cases, fluoroscopy‑based VATS procedures need to be performed, exposing patients and surgeons to radiation.

Both conventional and soft HW localization are simple procedures, as evidenced by the similarly high (>95%) technical success rates observed in the study groups, which translates to a lack of any clear differences in terms of their localization performance. However, the patients who underwent soft HW localization were exposed to lower levels of pain, which makes this techinque a more comfortable alternative to the conventional HW approach for CT‑guided GGN localization.

**TABLE 3:** Characteristics and outcomes of video-assisted thoracoscopic surgery

Parameter		Conventional hook-wire group	Soft hook-wire group	*P *value
Technical success of limited resection		94 (98.9)	88 (100)	>0.99
Additional lobectomy	Yes	27 (28.7)	26 (29.5)	0.9
	No	67 (71.3)	62 (70.5)	
Final diagnosis	Invasive adenocarcinoma	27 (28.4)	28 (31.8)	0.17
	Mini-invasive adenocarcinoma	46 (48.4)	32 (36.4)	
	Adenocarcinoma in situ	7 (7.4)	15 (17)	
	Precancerous lesion	8 (8.4)	4 (4.6)	
	Benign lesion	7 (7.4)	9 (10.2)	

Data are presented as number (percentage) of patients.

Complication rates in the soft HW group were significantly lower than in the conventional HW group, potentially owing to the rigid structure of the conventional HW materials. Such rigidity may cause more substantial pain and increase the risk of complications when the patients move their bodies. We found no significant differences in pneumothorax or lung hemorrhage rates between the study groups; however, this may be related to the limited sample sizes for analyses of individual complications.

Both conventional and soft HW localization exhibited relative high overall complication rates (48.9% and 32.9%) in this study, and these rates were higher than the overall complication rate (24.8%) of coil localization reported in a previous meta‑analysis.^19^ During coil localization, the coil tail remains on the surface of the pleura.^19^ In contrast, both conventional and soft HW should ideally penetrate the thoracic wall, with the tail remaining outside the thoracic wall. This may be the cause of the relative high complication rates associated with these techniques.

Relative to liquid‑ or coil‑based localization strategies, HW localization offers some advantages. Notably, the markers on the HW tail can aid operators in the intraoperative assessment of GGN depth, informing the selection of an appropriate resection range. Comparable resection rates were observed in the conventional and soft HW groups in this study, suggesting that the type of HW material does not impact the outcome of the VATS procedure.

Accurate preoperative CT‑guided localization of GGNs is important for preserving lung function in GGN patients undergoing VATS resection. As stated before, GGNs are not palpable. What is more, most of them exhibit a pathological stage below IA. In line with that, only 24.6% of the GGNs in this study were confirmed to be IAs, necessitating additional lobectomy. In the remaining 75.4% of cases, only limited resection was required.

There are some limitations to this study. Firstly, it was a retrospective analysis, and further prospective validation of the findings is vital. Secondly, the GGN diameters were different between the groups, potentially contributing to selection bias. Thirdly, the patients in the conventional and soft HW groups were treated during different periods; however, all procedures were performed by the same surgeons, potentially reducing the associated bias. Lastly, only univariable analysis was performed in this study.

## CONCLUSIONS 

In summary, both conventional and soft HW preoperative localization strategies can be successfully used to guide the VATS resection of pulmonary GGNs. However, soft HW localization exhibits a more favorable safety profile.
